# Additive prognostic value of serum calcium to the ESC risk stratification in patients with acute pulmonary embolism

**DOI:** 10.1186/s12959-023-00461-y

**Published:** 2023-02-15

**Authors:** Jiarui Zhang, Adila Ali, Yu Liu, Lige Peng, Jiaqi Pu, Qun Yi, Haixia Zhou

**Affiliations:** 1 Department of Respiratory and Critical Care Medicine, West China Hospital of Sichuan University, Chengdu, Sichuan China; 2 Cancer Hospital Affiliate to School of Medicine, Sichuan Cancer Hospital and Institution, Sichuan Cancer Center, UESTC, Sichuan Province, Chengdu, China

**Keywords:** Acute pulmonary embolism, Serum calcium, ESC prognostic algorithm, Risk stratification

## Abstract

**Background:**

Hypocalcemia has been shown to be involved in the adverse outcomes of acute pulmonary embolism (APE). We aimed to determine the incremental value of adding hypocalcemia, defined as serum calcium level ≤ 2.12 mmol/L, on top of the European Society of Cardiology (ESC) prognostic algorithm, for the prediction of in-hospital mortality in APE patients, which in turn could lead to the optimization of APE management.

**Methods:**

This study was conducted at West China Hospital of Sichuan University from January 2016 to December 2019. Patients with APE were retrospectively analyzed and divided into 2 groups based on serum calcium levels. Associations between hypocalcemia and adverse outcomes were assessed by Cox analysis. The accuracy of risk stratification for in-hospital mortality was assessed with the addition of serum calcium to the current ESC prognostic algorithm.

**Results:**

Among 803 patients diagnosed with APE, 338 (42.1%) patients had serum calcium levels ≤ 2.12 mmol/L. Hypocalcemia was significantly associated with higher in-hospital and 2-year all-cause mortality compared to the control group. The addition of serum calcium to ESC risk stratification enhanced net reclassification improvement. Low-risk group with serum calcium level > 2.12 mmol/L had a 0% mortality rate, improving the negative predictive value up to 100%, while high-risk group with serum calcium level ≤ 2.12 mmol/L indicated a higher mortality of 25%.

**Conclusion:**

Our study identified serum calcium as a novel predictor of mortality in patients with APE. In the future, serum calcium may be added to the commonly used ESC prognostic algorithm for better risk stratification of patients suffering from APE.

## Introduction

Acute pulmonary embolism (APE) is a common cause of hospital and mortality, which results in a heavy disease burden on both families and countries [1]. The clinical severity of APE is variable and ranges from minor symptoms to right ventricular dysfunction and cardiogenic shock [2, 3]. The early detection of high-risk patients represents an important step in prudent therapeutic decision making and reducing the risk of mortality. The European Society of Cardiology (ESC) prognostic algorithm is currently used in the risk assessment in patients with APE [4–6]. Patients were classified into low, intermediate-low, intermediate-high and high mortality risk groups based on the assessment of hemodynamic instability, clinical status, and laboratory indicators of APE severity, mostly related to the presence of right ventricular (RV) dysfunction [6, 7]. Low-and intermediate–low-risk APE patients can be candidates for home anticoagulant treatment or short hospital stays, intermediate–high-risk APE patients need additional close monitoring, and high-risk PE patients require urgent systemic thrombolysis or surgical embolectomy therapy to improve RV function [8].

Calcium is an important coagulation factor that participates in different cellular processes [9]. Hypocalcemia is a common biochemical abnormality and has been recognized as a prognostic marker of coronary heart disease, chronic kidney disease, acute myocardial infarction and gastrointestinal bleeding [10–13]. Furthermore, evidence is mounting that hypocalcemia is associated with short-term mortality after APE [14, 15]. A study of 4196 consecutive subjects revealed that serum calcium improved the simplified Pulmonary Embolism Severity Index (sPESI) score for risk stratification in patients with APE [16]. However, whether the assessment of serum calcium adds to the ESC risk stratification to produce greater power for predicting in-hospital and long-term mortality is unclear.

The aim of this study was to investigate the incremental value of adding serum calcium on top of the ESC-defined prognostic algorithm for the prediction of in-hospital and long-term mortality in patients with APE. We particularly sought to determine whether it permits the identification of high-risk patients with an even higher degree of safety.

## Methods

### Study design

This study was retrospectively conducted at West China Hospital of Sichuan University, China. The study enrolled consecutive adult inpatients diagnosed with APE from January 2016 to December 2019. The diagnosis of APE was based on the following criteria: (1) CTPA showing a segmental or proximal filling defect; or (2) the V/Q scan yielding high probability.

for pulmonary embolism. We excluded patients with incomplete initial clinical data. Patients with a diagnosis of hyperparathyroidism were also excluded for the impact on serum calcium. This study was approved by the Institutional Review Board of West China Hospital of Sichuan University, which waived the requirement for written informed consent because of the retrospective study design.

### Data collection

Data were obtained from the hospital’s computerized medical records, including demographic data, symptoms, vital signs, comorbidities and laboratory and radiographic findings. The presence of comorbidities was defined according to clinical diagnosis. Laboratory and radiographic data, including N-terminal-pro-B-type natriuretic peptide (NT-proBNP), troponin T, RV dilation (defined as the diameter ratio of right ventricle and left ventricle qualed or exceeded 1 by computed tomography (CT) or ultrasound), and pleural effusion were reviewed. The use of anticoagulant agents (low-molecular weight heparin (LMWH), fondaparinux, unfractionated heparin, vitamin K antagonists, or nonvitamin K antagonist oral anticoagulants), thrombolytic, interventional or surgical treatment was directed by the attending physician in accordance with clinical protocols based on ESC guidelines [6].

### Laboratory assessment of serum calcium

The assessment of serum calcium was routinely conducted for all of the enrolled patients within 24 h after admission. Levels of serum calcium were measured by Arsenazo III colorimetry (Beckman Coulter, Brea, America) using fresh samples, which had a functional sensitivity of 0.01 mmol/L. It was unadjusted for serum albumin and the reference interval of serum calcium for our laboratory ranged from 2.11 to 2.52 mmol/L. Hypocalcemia was defined as a serum calcium level ≤ 2.12 mmol/L [17].

### ESC risk stratification

The ESC prognostic algorithm for patients with APE was calculated using the following criteria: (i) signs of hemodynamic instability, RV overload and plasma troponin concentrations; and (ii) presence of comorbidity and any other clinical symptoms [6]. Patients were divided into three groups based on the ESC guidelines: low risk, intermediate risk (intermediate–low and intermediate–high risk), and high risk.

### Follow-up and outcomes

Each patient was followed up with a telephone conversation after admission until 2 years. The primary outcomes of the study was in-hospital and 2-year all-cause mortality, while the secondary outcomes were in-hospital APE-related mortality, intensive care unit (ICU) admission, respiratory failure, mechanical ventilation and length of stay.

### Statistical analysis

Continuous variables are reported as the means with standard deviations or medians with interquartile ranges and were compared using Student’s* t* test or the Mann–Whitney *U* test, while categorical variables are presented as percentages and were compared using the chi-square test. All parameters were compared between the hypocalcemia group and the control group. Univariable and multivariable Cox regression analyses were conducted to confirm the risk factors for mortality, which are presented as hazard ratios (HRs) with 95% confidence intervals (CIs). For survival analysis, Kaplan–Meier analysis and log-rank test were used to describe and compare mortality between the patients with serum calcium level ≤ 2.12 mmol/L and serum calcium level > 2.12 mmol/L in the population with APE. The changes in predicted risk reclassification were calculated for the evaluation of the improvement in prediction performance gained by adding the serum calcium to the ESC prognostic algorithm. A P value of less than 0.05 was considered to be statistically significant, and analyses were performed with SPSS software (IBM Corporation, Armonk, New York, United States).

## Results

### Baseline information

**Fig. 1:**
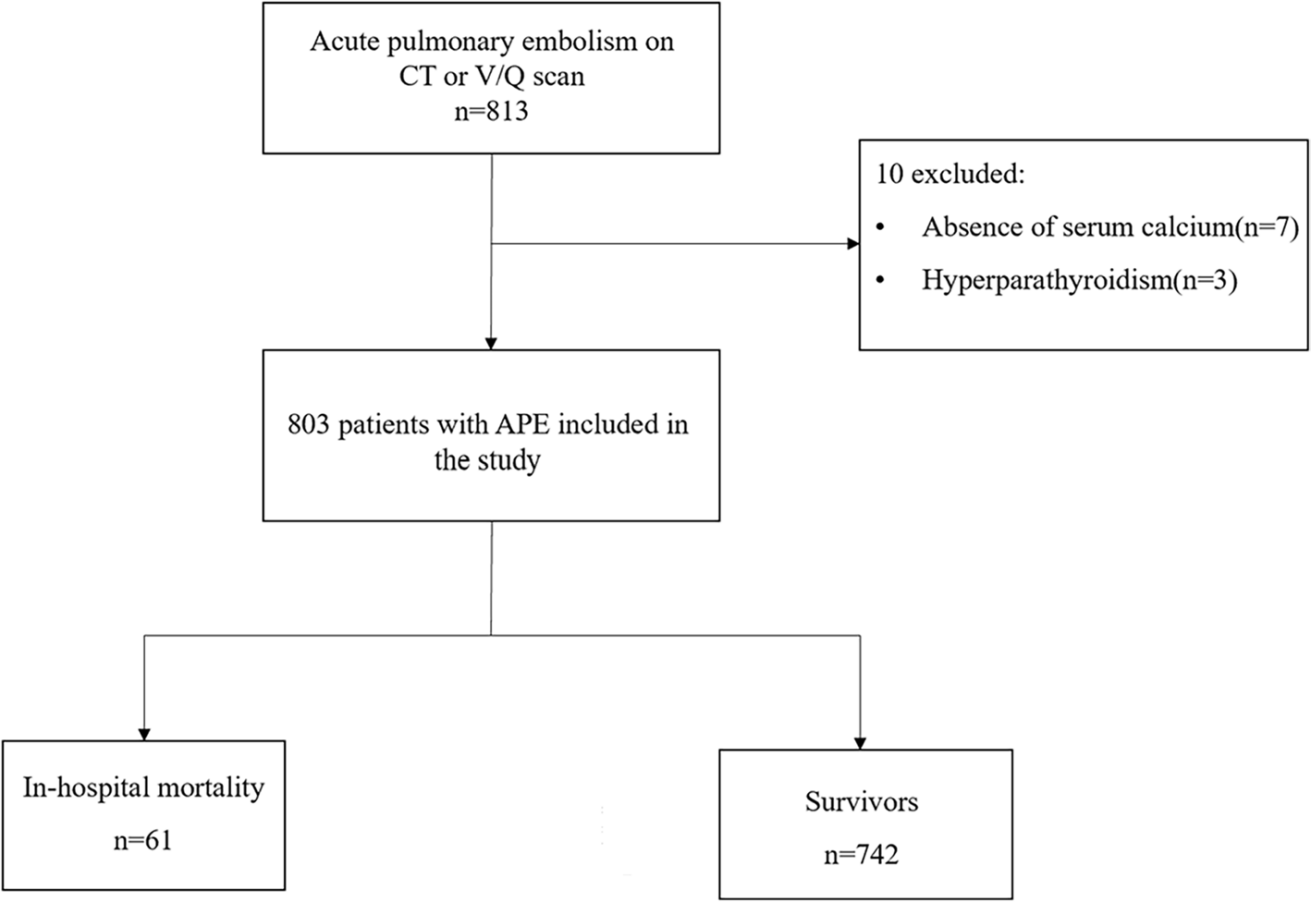
Patient inclusion flowchart

During the study period, a total of 813 patients were identified as having APE. Patients were excluded from the analyses: 7 patients did not have serum calcium measurements, and 3 patients had hyperparathyroidism. The final analysis included 803 patients, of whom 61 died and 742 survived (Fig. 1).

### Clinical characteristics of patients with APE and hypocalcemia

There were 338 patients with serum calcium levels ≤ 2.12 mmol/L (hypocalcemia group) and 465 with serum calcium levels > 2.12 mmol/L (control group). The demographic and clinical characteristics of patients with and without hypocalcemia are presented in Table 1. Compared with the control group, the hypocalcemia group showed a significantly higher percentage of males (222[65.7%] vs. 255[54.8%], *P* = 0.002) and smokers (110[32.5%] vs. 121[26.0%], P = 0.044). Additionally, dyspnea, hemoptysis, and leg pain or swelling were more frequently observed in the hypocalcemia group than in the control group, but the difference was not statistically significant. Patients admitted with hypocalcemia had lower systolic blood pressure, higher body temperature, and faster pulse rate and respiratory rate (both *P* < 0.05). According to the PESI and sPESI, high-risk patients comprised a significantly greater percentage of the hypocalcemia group than of the control group (*P* < 0.001 and *P* = 0.017). Except for active cancer and coronary heart disease, the data of comorbid conditions did not significantly differ between the 2 groups.

**Table 1:** Clinical characteristics of 803 patients with APE

Characteristics	Patients with hypocalcemia (serum calcium level ≤ 2.12 mmol/L, n = 338)	Patients without hypocalcemia (serum calcium level > 2.12 mmol/L, n = 465)	*P* value
Age(years)	60.16 ± 17.63	58.65 ± 15.92	0.213
Male sex	222(65.7)	255(54.8)	0.002
Smoking status			0.044
Smoker/ever smoker	110(32.5)	121(26.0)	
Non-smoker	228(67.5)	344(74.0)	
**Symptoms on admission**			
Dyspnea	243(72.3)	309(67.0)	0.110
Chest pain	108(32.0)	160(34.6)	0.428
Hemoptysis	86(25.4)	114(24.7)	0.804
Syncope	39(11.5)	59(12.7)	0.607
Leg pain or swelling	119(35.2)	137(29.5)	0.088
**Physical examination findings**			
Systolic blood pressure (mmHg)	122.00 ± 20.82	125.42 ± 20.48	0.021
Pulse rate (b.p.m)	92.20 ± 17.64	85.97 ± 16.28	< 0.001
Respiratory rate (/min)	21.33 ± 3.57	20.55 ± 2.48	0.001
Temperature ( °C)	36.72 ± 0.63	36.56 ± 0.46	< 0.001
**Predisposing or comorbid condition**			
Chronic pulmonary disease	119(35.2)	135(29.0)	0.063
Active Cancer	55(16.3)	117(25.2)	0.002
Hypertension	82(24.3)	123(26.5)	0.482
Pulmonary tuberculosis	21(6.2)	22(4.7)	0.361
Diabetes	29(8.6)	37(9.6)	0.694
Coronary heart disease	12(3.6)	37(8.0)	0.010
Chronic renal insufficiency	15(4.4)	16(3.4)	0.473
PESI			
I-II	138(40.8)	245(52.7)	0.001
III	93(27.5)	125(26.9)	0.842
IV-V	107(31.7)	95(20.4)	< 0.001
sPESI			0.017
High risk	296(87.6)	378(81.3)	
Low risk	42(12.4)	87(18.7)	
**Laboratory and radiologic findings**			
Elevated cTnT	133(39.3)	165(35.5)	0.263
Elevated NT-pro BNP	178(52.7)	199(42.8)	0.006
Right ventricle dilation on CT	110(48.5)	132(38.8)	0.023
Pleural effusion	244(72.6)	257(55.4)	< 0.001
**Clinical outcome**			
In-hospital mortality	36(10.7)	25(5.4)	0.005
In-hospital APE-related mortality	15(4.4)	10(2.2)	0.065
ICU admission	20(5.9)	19(4.1)	0.233
Systemic thrombolysis	5(1.5)	5(1.1)	0.750
Respiratory failure	85(25.1)	70(15.1)	< 0.001
Mechanical ventilation	24(7.1)	18(3.9)	0.042
Length of hospital stay, days	15(9–23)	14(9–22)	0.516
720-day mortality	83(24.6)	84(18.1)	0.025

Abbreviations: *PESI* Pulmonary embolism severity index, *sPESI* Simplified pulmonary embolism severity index, NT-pro-BNP = N-terminal pro-B–type natriuretic peptide, *CT* Computed tomography, *ICU* Intensive care unit

Note: Chronic lung disease included chronic obstructive pulmonary disease,bronchiectasis, bronchial asthma, pulmonary tuberculosis and idiopathic pulmonary fibrosis. Chronic renal insufficiency was defined as glomerular filtration rate < 60 ml/min because of chronic kidney disease(nephrotic syndrome, chronic glomerulonephritis, lupus nephritis and diabetic nephropathy)

### Laboratory and computed tomography findings in patients with APE and hypocalcemia

Patients with hypocalcemia had higher levels of NT-proBNP (*P* = 0.006), and there were no significant differences between groups in terms of troponin T. RV dilation was significantly more common (110 [48.5%] vs. 132 [38.8%], *P* = 0.023), and pleural effusion tended to be more common (244 [72.6%] vs. 257 [55.4%], *P* < 0.001) in the hypocalcemia group than in the control group.

### Hypocalcemia and clinical outcome

A total of 61 (7.6%) patients died during hospitalization, and 25 (3.1%) deaths were adjudicated as APE-related. The in-hospital and 2-year all-cause mortality was significantly higher in the hypocalcemia group than in the control group (both *P* < 0.05) (Table 1). The presence of hypocalcemia was associated with a higher rate of respiratory failure (25.1% vs. 15.1%, *P* < 0.001) and mechanical ventilation (7.1% vs. 3.9%, *P* = 0.042). However, there were no significant differences between groups in terms of APE-related mortality, ICU admission, systemic thrombolysis, or length of hospital stay (all *P* > 0.05).

### Predictors of mortality and survival analysis

All patients included in the study were divided into a death group (*n* = 61) and a survival group (*n* = 742). The results of univariable hazard risk analysis for the prediction of in-hospital all-cause mortality are presented in Table 2. The multivariable hazard risk analysis showed that independent predictors of fatal outcome were age, male sex, systolic blood pressure < 100 mmHg, pulse rate ≥ 110 beats/min, active cancer, chronic renal insufficiency, and serum calcium level ≤ 2.12 mmol/L(Table 3).

**Table 2:** Univariable hazard risk predictors of in-hospital all-cause mortality

Parameter	Hazard risk	95% confidence interval	*P*-Value
Age(years)	1.033	1.015–1.051	< 0.001
Male sex	2.162	1.187–3.936	0.012
Systolic blood pressure < 100 mmHg	3.964	2.172–7.233	< 0.001
Pulse rate ≥ 110beats/min	4.79	2.856–8.033	< 0.001
Active Cancer	2.214	1.305–3.755	0.003
Diabetes	3.008	1.652–5.477	< 0.001
Chronic renal insufficiency	3.626	1.793–7.334	< 0.001
Right ventricle dilation on CT	1.83	0.936–3.580	0.078
Pleural effusion	1.781	0.963–3.294	0.066
Serum calcium level ≤ 2.12 mmol/L	1.874	1.124–3.125	0.016

Abbreviations: *CT* Computed tomography

Note: Chronic renal insufficiency was defined as glomerular filtration rate < 60 ml/min because of chronic kidney disease(nephrotic syndrome, chronic glomerulonephritis, lupus nephritis and diabetic nephropathy)

**Table 3:** Independent multivariable hazard risk predictors of in-hospital all-cause mortality

Parameter	Hazard risk	95% confidence interval	*P*-Value
Age(years)	1.022	1.004–1.039	0.016
Male sex	2.011	1.089–3.716	0.026
Systolic blood pressure < 100 mmHg	3.425	1.809–6.485	< 0.001
Pulse rate ≥ 110beats/min	4.504	2.655–7.642	< 0.001
Active Cancer	2.866	1.605–5.119	< 0.001
Diabetes	1.922	0.930–3.973	0.078
Chronic renal insufficiency	2.523	1.096–5.810	0.03
Serum calcium level ≤ 2.12 mmol/L	1.764	1.006–3.095	0.048

Note: Chronic renal insufficiency was defined as glomerular filtration rate < 60 ml/min because of chronic kidney disease(nephrotic syndrome, chronic glomerulonephritis, lupus nephritis and diabetic nephropathy)

**Fig. 2:**
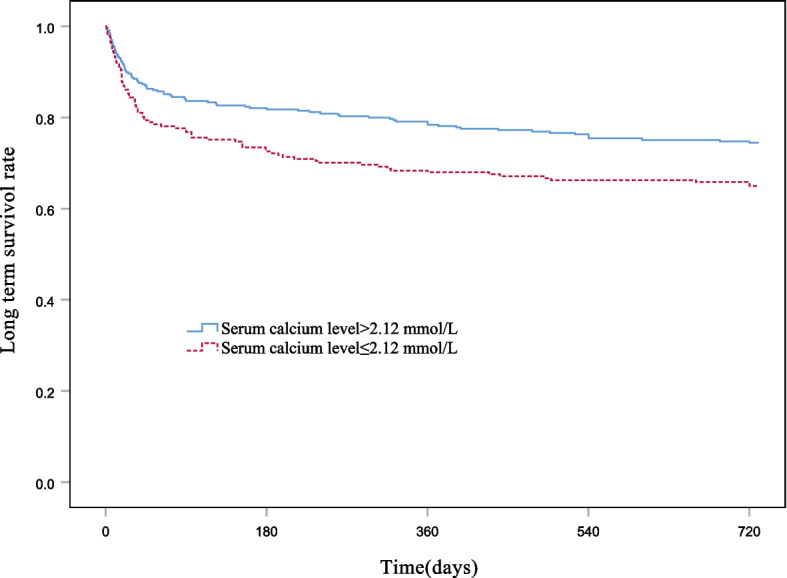
Kaplan–Meier curves of overall survival in patients with APE. The event was defined as 720-day mortality, and patients were divided into hypocalcemia and control groups according to the level of serum calcium (*P* = 0.012)

The log-rank test was used to compare the difference in survival between the hypocalcemia and control groups, while the Kaplan–Meier method was used to draw a survival curve. The results revealed a significant difference in 2-year all-cause mortality between the two groups (*P* = 0.005) (Fig. 2).

### Combining ESC Risk Stratification and Serum Calcium for Prognostic Assessment

**Fig. 3:**
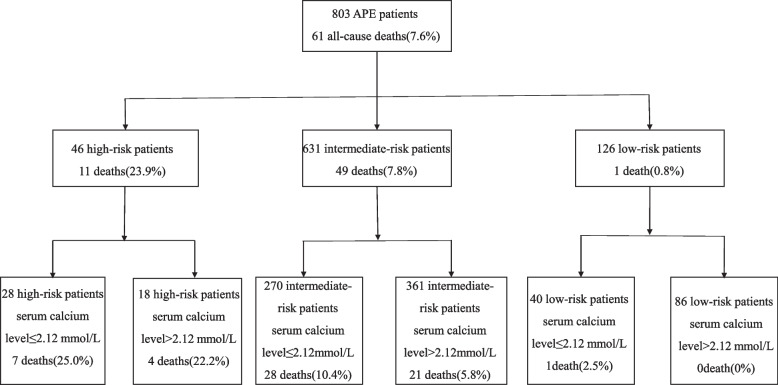
The inclusion of serum calcium assessment on admission into the in-hospital prognostic model based on the ESC guidelines significantly improved the predictive accuracy of the risk evaluation in the APE

Forty-six (5.7%) patients presented with hemodynamic instability and formed a high-risk group, whereas the remaining 757 patients were normotensive on admission. Eleven deaths occurred in the high-risk APE group (mortality 23.9%), and a serum calcium level ≤ 2.12 mmol/L on admission indicated 28 patients with 25.0% mortality, while a serum calcium level > 2.12 mmol/L indicated 22.2% mortality. In the intermediate-risk group, the in-hospital mortality was 7.8%. Patients with hypocalcemia had a higher observed mortality rate than patients with normal serum calcium (10.4% vs. 5.8%). One (0.8) death was reported in the low-risk group, and a serum calcium level > 2.12 mmol/L on admission was present in a group of 126 APE patients with a mortality rate of 0% (Fig. 3).

## Discussion

This study investigated the prognostic significance of hypocalcemia, defined as a serum calcium level ≤ 2.12 mmol/L, for APE. Our results showed that lower serum calcium was associated with worse in-hospital and long-term mortality. Importantly, the present study might be the first to reveal that the combined use of serum calcium and the ESC prognostic algorithm was better able to predict in-hospital death compared with ESC risk stratification alone. Furthermore, early assessment of serum calcium might accurately identify high-risk patients with APE.

The ESC prognostic algorithm was developed for the early risk assessment of patients with APE, which represented an important step in therapeutic decision making [8]. Moor et al. reported that the area under the receiver operating characteristic curve (AUC) predictive value of the 2019 ESC algorithm for 30-day mortality was 0.636 [18]. In a prospective cohort study, the risk of death in patients at “intermediate-high” and “intermediate-low” risk according to the ESC model was similar, and risk stratification in patients at intermediate risk requires further improvement [19]. Therefore, the categorization of patients with APE based on RV dysfunction, elevated troponins and sPESI seems not sufficiently efficient.

Hypocalcemia is one of the most common electrolyte disturbances and may potentially impact virtually any organ and system [17]. There is evidence that serum calcium independently predicts mortality in acute PE. In a study including 2017 nationwide inpatients with APE, Murthi et al. found that patients with hypocalcemia had higher in‑hospital mortality and complications than those without hypocalcemia, along with a longer length of stay [15]. Wang et al. reported that hypocalcemia was an independent predictor of 30-day mortality following APE [14]. In the present study, the existence of hypocalcemia was an independent predictor of all-cause in-hospital and long-term mortality of APE, which reported that hypocalcemia showed better prognostic predictive performance and could be a novel marker for predicting poor prognosis of APE. These studies encouraged us to add serum calcium to the ESC prognostic algorithm, with the aim of improving its prognostic value.

This study showed that serum calcium assessment improved the accuracy of the current ESC risk stratification strategy in identifying patients with APE at increased risk of death. Our results strengthen those described by Yang et al., who proposed a new prognostic assessment model combining the sPESI risk score with serum calcium, which had higher performance than the PESI and sPESI [16]. In this study, a serum calcium level ≤ 2.12 mmol/L in patients with low risk identified a group with a mortality of 2.5%, improving the negative predictive value up to 100%, while in high-risk patients, it indicated a high early mortality of 25%. Some ESC-defined intermediate-risk APE patients were accurately stratified as high and low risk after adding serum calcium to the model. Compared with the ESC prognostic algorithm alone, serum calcium assessment significantly improved the current risk stratification according to the ESC guidelines.

Our study clarified that hypocalcemia was significantly associated with a higher PESI and sPESI score, higher blood levels of NT-proBNP, and higher rates of RV dilation and pleural effusion compared to those without hypocalcemia. All of these clinical prediction rules are known strong predictors of short-term and long-term prognosis after APE [20–24]. Based on these findings, we assumed that hypocalcemia influences the short-term and long-term prognosis of APE. Mechanisms by which hypocalcemia has been hypothesized to be associated with mortality include APE with a high risk of causing proinflammatory cytokine release, inhibiting parathyroid hormone release, causing cellular redistribution of calcium, and consequently precipitating hypocalcemia [15]; hypocalcemia is associated with cardiac dysfunction and hypotension [25]; and vitamin D deficiency is considered to be one of the most common causes of hypocalcemia and contributes to poor prognosis in patients with cardiopulmonary disease [26, 27].

Based on the findings of this study, some suggestions for management and treatment were given about APE. The presence of hypocalcemia has an important prognostic impact for APE and might help predict short-term and long-term mortality, which should be closely monitored for the benefits of patients with APE in the hospital and after discharge. On the other hand, serum calcium assessment may be added to the ESC prognostic algorithm to acquire higher predictive power for in-hospital mortality.

Several limitations of the present study require consideration. Firstly, these findings come from a single-center, retrospective study and cannot be extrapolated to other APE patients. Validation with a prospective population in a well-designed multicenter study is needed. Secondly, we also did not collect data on the use of blood products, which may affect serum calcium levels. Therefore, we could not determine the correlation between these factors and mortality. Thirdly, it is unclear if hypocalcemia in itself affects the prognosis, or if it is a surrogate marker for another factor (e.g. vitamin D deficiency). Fourthly, the prevalence of hypocalcemia in our study was much higher than that reported in previous studies, in which it reported prevalence rates of 0.8% [15]. However, this was in accordance with the findings by PUMCH cohort study conducted in China recently, which reported that the prevalence of hypocalcemia was 49.48% in acute pulmonary embolism [14]. In addition to the possible effect of the different patient populations investigated, we believed that this large difference in the cutoff value of serum calcium levels among the studies can mainly be attributed to differences in the prevalence of hypocalcemia in patients with APE. Fifthly, we only explored the relationship between serum calcium levels on admission and mortality in patients with APE. Serum calcium levels change as the disease progresses, and we did not report the relationship between dynamic changes in serum calcium levels and prognosis in patients with APE.

## Conclusion

Hypocalcemia present on admission in 42.1% of patients with acute PE indicated worse prognosis. Serum calcium ≤ 2.12 mmol/L not only independently predicted higher in-hospital and long-term all-cause mortality but also, when added to the current ESC risk stratification algorithm, improved the identification of both low- and high-risk patients. Therefore, serum calcium assessment may be implemented in the risk assessment of APE.

## Data Availability

The datasets used and/or analyzed during the current study are available from the corresponding author on reasonable request.

## Abbreviations

APE: Acute pulmonary embolism
ESC: European society of cardiology
RV: Right ventricular
PESI: Pulmonary embolism severity index
sPESI: Simplified pulmonary embolism severity index
NT-pro BNP: N-terminal pro-B–type natriuretic peptide
CT: Computed tomography
LMWH: Low-molecular weight heparin
ICU: Intensive care unit
HR: Hazard ratios
CI: Confidence interval
AUC: Area under the receiver operating characteristic curve
